# Proteomic analysis of haem-binding protein from *Arabidopsis thaliana* and *Cyanidioschyzon merolae*

**DOI:** 10.1098/rstb.2019.0488

**Published:** 2020-05-04

**Authors:** Takayuki Shimizu, Rintaro Yasuda, Yui Mukai, Ryo Tanoue, Tomohiro Shimada, Sousuke Imamura, Kan Tanaka, Satoru Watanabe, Tatsuru Masuda

**Affiliations:** 1 Graduate School of Arts and Sciences, The University of Tokyo, Komaba, Meguro-ku, Tokyo 153-8902, Japan; 2 Department of Bioscience, Tokyo University of Agriculture, Setagaya-ku, Tokyo 156-8502, Japan; 3 School of Agriculture, Meiji University, Kawasaki-shi, Kanagawa 214-8571, Japan; 4 Laboratory for Chemistry and Life Science, Institute of Innovative Research, Tokyo Institute of Technology, Yokohama-shi, Kanagawa 226-8503, Japan

**Keywords:** retrograde signal, haem, chloroplast, nucleoproteins, proteomics

## Abstract

Chloroplast biogenesis involves the coordinated expression of the plastid and nuclear genomes, requiring information to be sent from the nucleus to the developing chloroplasts and vice versa. Although it is well known how the nucleus controls chloroplast development, it is still poorly understood how the plastid communicates with the nucleus. Currently, haem is proposed as a plastid-to-nucleus (retrograde) signal that is involved in various physiological regulations, such as photosynthesis-associated nuclear genes expression and cell cycle in plants and algae. However, components that transduce haem-dependent signalling are still unidentified. In this study, by using haem-immobilized high-performance affinity beads, we performed proteomic analysis of haem-binding proteins from *Arabidopsis thaliana* and *Cyanidioschyzon merolae*. Most of the identified proteins were non-canonical haemoproteins localized in various organelles. Interestingly, half of the identified proteins were nucleus proteins, some of them have a similar function or localization in either or both organisms. Following biochemical analysis of selective proteins demonstrated haem binding. This study firstly demonstrates that nucleus proteins in plant and algae show haem-binding properties.

This article is part of the theme issue ‘Retrograde signalling from endosymbiotic organelles’.

## 1. Introduction

Haem serve as cofactors of haemoproteins in various organelles that function in mitochondria respiratory and chloroplast photosynthetic electron transport chains, and in the detoxification of reactive oxygen species and xenobiotics, as well as in oxygen storage and transport [1]. In addition, haem has been proposed to be a regulatory factor in control of transcription and intercellular signalling in yeast and animals [2,3].

The haem biosynthetic pathway begins with the synthesis of 5-aminolevulinic acid, the universal precursor of all tetrapyrroles. In photosynthetic organisms, the tetrapyrrole biosynthesis pathway branches into chlorophyll or haem synthesis, where the metabolite protoporphyrin IX (Proto) is the substrate of two structurally different metal chelatases. The Mg-chelatase converts Proto to Mg-protoporphyrin IX (MgProto) and ferrochelatase (FC) inserts Fe^2+^ into Proto to form haem (protohaem). All higher plants analysed so far possess two genes encoding FC (*FC1* and *FC2*), which show differential tissue-specific and development-dependent expression profiles, such that *FC2* is light-dependent and mainly expressed in photosynthetic tissues, whereas *FC1* is stress-responsive and ubiquitously expressed in all tissues [4,5]. Concerning the subcellular localization, the main FC activity is detected in chloroplasts and has very low activity in mitochondria [6,7], although the possibility of mitochondrial localization of FC cannot be excluded [8]. In the green algae *Chlamydomonas reinhardtii*, a single *FC* encodes a plastid-localized FC protein [9], while in the red algae *Cyanidioschyzon merolae,* FC is only found in mitochondrial extracts [10]. These results suggest that in Streptophyta and Cholorphyta, the dominant plastid FC activity supplies haem for the plastid as well as other organelle-localized haemoproteins, while distinct mitochondrial haem biosynthesis is employed in Rhodophyta. In these photosynthetic organisms, the function of haem is not limited to their roles as prosthetic groups, but they are also proposed to serve as signalling molecules [11,12].

Chloroplast biogenesis involves the coordinated expression of the plastid and nuclear genomes, requiring information to be sent from the nucleus to the developing chloroplasts and vice versa. The latter is achieved through plastid-to-nucleus (retrograde) signalling pathways in which plastids send a signal to regulate various physiological phenomena, such as photosynthesis-associated nuclear genes (PhANGs) expression [11], and cell cycle coordination [13], depending on their developmental and functional states. Genetic and biochemical analyses of this pathway suggest a major role for haem in retrograde signalling. In *Arabidopsis thaliana*, mutations affecting chloroplast function or treatments with inhibitors such as norflurazon (NF) or lincomycin (Lin) result in the strong repression of many PhANGs. Characterization of *genomes uncoupled* (*gun*) mutants in which the expression of the nuclear gene *Lhcb* is maintained following chloroplast damage using NF treatment [14] suggests the involvement of tetrapyrroles in retrograde signalling. Among the original five *gun* mutants described, *gun2* and *gun3* lack a functional haem oxygenase 1 and phytochromobilin synthase [15], and *gun4* and *gun5* are mutants of the regulator [16] and the H subunit of Mg-chelatase [15], respectively. More recently, the identification of a dominant *gun6* mutant with increased FC1 activity [17] restores PhANGs expression even when chloroplast development is blocked. These data suggest that increased flux through the FC1-producing haem may act as a signalling molecule that control PhANGs as a retrograde signal in *A. thaliana*.

Signalling function of haem is not limited in higher plants. In *Ch. reinhardtii*, haem along with MgProto has been proposed as a signalling molecule that may substitute for light [18]. Analysis of the transcriptome in *Ch. reinhardii* showed that the expression of hundreds of genes was affected by exogenous haem treatment, but only a few of them were associated with photosynthesis [19]. In *Cy. merolae*, abscisic acid (ABA) induced haem-scavenging tryptophan-rich sensory protein-related protein (TSPO), resulting in inhibition of the cell cycle G1/S transition [20]. Because the ABA-dependent inhibition of DNA replication was negated by addition of exogenous haem, it is proposed that ABA and haem have regulatory role in algal cell cycle initiation [20].

As described above, for assembly of holoproteins, haem synthesized in plastids of *A. thaliana* and *Ch. reinhardtii* or in mitochondria of *Cy. merolae* should be transported to the appropriate cellular organelles, such as peroxisome, endoplasmic reticulum (ER) and nucleus. However, compared with bacteria, yeast and animals, the mechanism of haem trafficking from plastid or mitochondria to other organelles in photosynthetic organisms is still largely unknown. For membrane transport, involvement of the membrane-bound ABC (ATP-binding cassette) transporters and TSPO, was proposed in animal cells [11]. In fact, ABC transporters, such as ABCB6 and ABCG2/BCRP, are involved in tetrapyrrole trafficking in mammalian cells [21,22] and *Arabidopsis* vacuolar ABC transporters AtMRP1–3 can transport chlorophyll catabolites to the vacuole during chlorophyll degradation [23]. In addition, homologues of TSPO in *A. thaliana* [24] and *Cy. merolae* [20] showed haem-binding properties and were induced by ABA treatment. However, the TSPO was localized to the secretary pathway [24]. In addition, because haem is poorly soluble in aqueous solutions under physiological conditions, involvement of haem carrier proteins was proposed [11]. The cytosolic p22HBP/SOUL protein which showed high affinity for haem was identified in animal cells [11]. A homologue of p22HBP/SOUL in *A. thaliana* was identified, which showed high affinity for haem, although its detailed function is unknown [25].

To elucidate the molecular mechanism of haem trafficking and signalling role, it is important to identify its molecular target(s). For this purpose, we have developed haem-immobilized high-performance affinity beads that allow single-step affinity purification of drug target proteins from crude cell extracts [26]. Here, we performed affinity purification of haem-binding proteins from *A. thaliana* and *Cy. melorae* cell extracts. Comparative analysis of these evolutionarily distant photosynthetic organisms will allow us to discuss shared features of the haem-binding proteins, as well as their diversity. Following proteomic analysis successfully identified possible candidate proteins that bind to haem. Our data suggest that haem is actually transferred into the nucleus and regulate not only transcription but also RNA metabolism and chromatin remodelling.

## 2. Material and methods

### (a) Preparation of haemin-immobilized ferrite-glycidyl methacrylate bead

Magnetic ferrite-glycidyl methacrylate (FG) beads (5 mg) (Tama Seiki), were incubated with 10 mM 1-hydroxybenzotriazole, 10 mM 1-ethyl-3-(3-demithyl-aminopropyl)-carbodiimide HCl and 2 mM haemin in *N,N*-dimethylformamide for 4 h at room temperature. Unreacted residues were masked using 20% carbonic anhydride in *N,N*-dimethylformamide, and the resulting beads were stored at 4°C.

### (b) Plant material and growth conditions

*Arabidopsis thaliana* wild-type (WT) was the Columbia-0 (Col-0) ecotype. Seeds were sown onto Murashige and Skoog medium supplemented with 1% (w/v) agar (pH 5.8) and incubated in white light (100 µmol m^−2^ s^−1^) for 2 h to induce germination. For protein extraction, seedlings were then grown for four weeks under continuous white light at 22°C. *Cyanidioschyzon merolae* 10D was grown at 40°C in MA2 medium under bubbling with 2% CO_2_ and continuous illumination (50 µmol m^−2^ s^−1^) [10].

### (c) Affinity purification of haemin-binding proteins

Haemin-immobilized beads (0.5 mg) were equilibrated with 0.5% NP-40 lysis buffer (50 mM Tris–HCl (pH 8.0), 150 mM NaCl and 0.5% NP-40). Four-week-old *A. thaliana* seedlings (1.5 g) or *Cy. merolae* cells were harvested, ground into powder in liquid nitrogen, and then suspended in 5 ml of KCl lysis buffer (100 mM KCl, 12.5% glycerol, 20 mM HEPES-NaOH (pH 7.9), 1 mM MgCl_2_, 0.2 mM CaCl_2_, 0.2 mM EDTA, 0.1% NP-40, 1 mM DTT, 0.2 mM PMSF). Protein concentration of extracts was determined with RC/DC kit (BioRad, CA, USA). Extracts containing 1 mg of proteins were incubated with the 0.5 mg of beads for 2–4 h at 4°C. The beads were washed three times with 0.5% NP-40 lysis (or KCl) buffer, and bound proteins were eluted with the Laemlli SDS sample buffer. Eluted samples were separated by SDS–PAGE and detected by silver staining using a Pierce silver stain kit (Thermofisher Scientific, MI, USA).

### (d) Identification of haem-binding proteins

The haem-bound proteins were subjected to in trypsin digestion (in-solution tryptic digestion and guanidination kit, Thermofisher Scientific, MI, USA) and purified with C_18_ column tip. Peptides were analysed with matrix-assisted laser desorption ionization time-of-flight mass spectrometer (MALDI-TOF-MS, Bruker Daltonics, MI, USA) coupled with high-performance liquid chromatography. Data were analysed by the Mascot algorithm to identify proteins corresponding to the peaks.

### (e) Expression and purification of recombinant proteins

For *A. thaliana* candidate proteins (At3g09650 and At5g55760), DNA fragments were polymerase chain reaction (PCR)-amplified using respective RAFL clones [27] as templates with appropriate primer sets (electronic supplementary material, table S1). pET24 vector (Novagen) was also PCR amplified. After pre-culture in Luria-Bertani (LB) medium containing 50 µg ml^−1^ kanamycin, proteins were induced by adding 1 mM isopropyl β-D-1-thiogalactopyranoside (IPTG) at 37°C for 3 h. For *Cy. merolae* candidate proteins (CMJ203C and CML100C), pETNH or pColdTF vector and genes were PCR-amplified using appropriate primer sets (electronic supplementary material, table S1). The obtained gene fragments and vectors were cloned using an InFusion cloning kit (TaKaRa, Shiga, Japan). The resulting plasmids were introduced into *Escherichia coli* strain BL21(DE3) or Rosetta 2(DE3)pLys competent cell (Merck Millipore, MI, USA). For expressing *E. coli* trigger factor (TF) as negative control, pColdTF vector (TaKaRa, Shiga, Japan) was also introduced into the Rosetta strain. The His-tagged fusion proteins were expressed as described previously [28].

*Escherichia coli* cells expressing recombinant proteins (500 ml LB medium) were suspended in 10 ml of Lysis buffer (20 mM Tris–HCl, pH 8.0, 500 mM NaCl, 10% glycerol, 5 mM imidazole) and disrupted by sonication. After centrifugation (10 000*g*, 30 min, 4°C), the soluble fraction was passed through a 0.45 µm filter membrane and subjected to 1 ml of HisTrap column (GE Healthcare, IL, USA) equipped in AKTA Start (GE Healthcare). After washing with 40 ml of Lysis buffer, the His-tag protein was eluted with a linear gradient of imidazole concentration (5–500 mM). Fractions containing purified proteins were collected and dialysed with buffer containing 20 mM Tris–HCl (pH 8.0), 500 mM NaCl, 6% glycerol.

### (f) Haem-binding assay

*Escherichia coli* cell pellets expressing candidate proteins were suspended in KCl buffer and sonicated using a Branson Sonifier (Branson Instruments, CT, USA). After centrifugation, soluble extract was mixed with haemin-agarose beads (Sigma-Aldrich, MO, USA) and used for the affinity purification as described above. Eluted samples were separated by SDS–PAGE and detected by western blot analysis using anti-His antibody. The cell extract expressing *A. thaliana* p22HBP [25] and TF proteins were used for the positive and negative control, respectively. For the haemin-competition assay, haemin solution, dissolved in 10 mM KOH, was added to the cell extract. After incubation for 4 h, the cell extracts were used for the affinity purification assay using haemin-agarose beads and analysed by western blot analysis using anti-His-tag antibodies. For spectrophotometric assay, purified proteins were mixed with equal or threefold concentration of haemin solution. Ultraviolet (UV)–visible absorbance spectra of haemin and haemin–protein complexes were taken in a Ultrospec 2100 pro spectrophotometer (GE Healthcare, IL, USA). For spectral changes assays, SRT1 (1 µM) and CML100C (10 µM) proteins were subjected to UV–visible absorbance spectroscopy, using 50 mM Tris–HCl, pH 8.0 as blank. Spectra were recorded between 300 and 700 nm using a 1 cm path length cuvette, in a haemin concentration range of 0–4 µM for SRT1 and 0–25 µM for CML100C. Difference spectra were obtained by subtracting the buffer spectrum from that of the protein–haemin complex. The concentration-dependent spectral studies for the different haemin were performed at least three times. The emergence of a red-shifted peak was fitted with the Origin software (OriginPro8 software; OriginPro Corporation, MA, USA). Data were analysed using nonlinear regression assuming one-site binding model.

### (g) Sirtuin assay

The SRT1 activity assay was performed as described in the manufacturer's protocol of the SIRT-Glo assay (Promega) using 0.3 µM of purified SRT1.

## 3. Results

### (a) Proteomic analysis of metal-tetrapyrrole binding proteins

**Figure 1.**
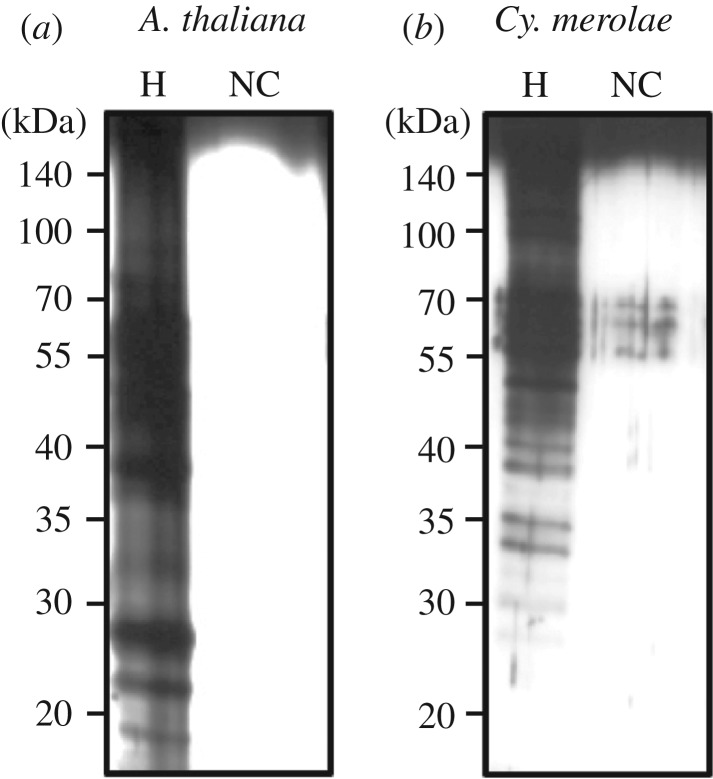
Identification of haem-binding proteins from *A. thaliana* and *Cy. merolae*. Bound fractions of cell extract from *A. thaliana* (*a*) and *Cy. merolae* (*b*) were eluted from haem-immobilized FG beads with the SDS sample buffer and separated by SDS–PAGE. Proteins were visualized by silver staining.

To purify haemin-binding proteins, we performed affinity purification using FG beads [26]. Haemin was covalently conjugated to the beads and incubated with extracts from *A. thaliana* and *Cy. merolae* cells. After extensive washing, bound proteins were eluted with the SDS sample buffer, and the eluate fractions were subjected to SDS gel electrophoresis and silver staining. As shown in figure 1, several bands were detected in fraction from haemin-immobilized beads, while almost no band was observed in beads without ligand showing negligible non-specific binding of proteins to the FG beads. These proteins were subjected to proteolytic digestion and MALDI-TOF-MS spectrometry. As a result, we identified 10 proteins from *A. thaliana* (table 1) and 10 proteins from *Cy. merolae* (table 2).

**Table 1.** Peptide identification for haem-binding proteins in *A. thaliana*.

description	protein accession numbers	putative localization	protein molecular size (kDa)	digest matches score (Mascot score)	peak no.	meas. *m/z*	ppm	positions	peptide sequence^a^
outer envelope pore protein 16–3	At2g42210	chloroplast/mitochondria	17.0	41.6	peak 3	718.295	−32.71	2–7	DPAEMR
					peak 5	870.559	91.284	42–48	DVPRVER
					peak 7	1930.938	−1.204	140–154	VDNGREYYPYTVEKR
PAUSED, a homologue of exportin-T	At1g72560	nucleus	111.5	30.8	peak 2	613.332	−56.41	704–708	VEPLR
					peak 5	870.559	49.444	704–710	VEPLRSK
					peak 6	1888.938	20.229	397–412	NNLNSLDKTGLEEEDR
					peak 7	1930.938	−34.844	300–316	VSALLTGYAVEVLE**C**HK
transcription factor bHLH110	At1g27660	nucleus	49.6	29.2	peak 2	613.332	−33.685	388–392	NRPGK
					peak 3	718.295	−73.124	312–318	AGENASK
					peak 6	1888.938	26.17	118–134	EELSSSTISDHQEGISK
protein kinase PINOID 2	At2g26700	nucleus	59.3	27.3	peak 3	718.295	−22.414	400–405	GDNNEK
					peak 4	842.52	−30.461	406–412	TLVNILK
					peak 6	1888.938	−40.444	475–489	SIKPPWVPKEETSHK
					peak 7	1930.938	−20.503	496–510	SVNYYLPPRFMMSRK
ABC transporter G family member 23	At5g19410	chloroplast envelope	70.0	25.5	peak 3	718.295	−73.102	163–167	EREER
					peak 4	842.52	72.554	618–624	KASKSTH
					peak 8	1946.958	−36.501	272–289	GSVIHLGSLEHLEDSIAK
NAD-dependent protein deacetylase SRT1	At5g55760	nucleus	52.6	24.6	peak 4	842.52	29.304	239–244	TPKDKK
					peak 5	870.559	91.284	76–82	EGKDLPK
					peak 8	1946.958	-49.43	274–290	IDLFQIILTQSISGDQR
pentatricopeptide repeat-containing protein	At3g09650	chloroplast	174.0	24.5	peak 1	569.305	95.943	496–499	GY**C**K
					peak 5	870.559	7.59	2907–303	IIDKGIK
					peak 6	1888.938	19.065	758–772	FWLGLPNSYYGSEWK
					peak 8	1946.958	2.516	338–352	DL**C**KVLRE**C**NAEDLK
Asp–Glu–Ala–Asp (DEAD)-box ATP-dependent RNA helicase	At2g35920	nucleus	111.0	23.9	peak 2	613.332	−56.41	370–374	DLLPR
					peak 3	718.295	−57.469	128–133	ADLDER
					peak 4	842.52	−43.806	150–156	KLGSLLK
					peak 8	1946.958	−68.106	674–691	ALQPPDALAVENAIELLK
calcium-dependent protein kinase 32	At3g57530	other	60.9	23	peak 4	842.52	−57.15	93–98	SILKKK
					peak 5	870.559	49.444	199–205	KETAPLK
					peak 7	1930.938	−32.749	101–116	TAVDIEDVRREVEIMR
transcription factor BOA	At5g59570	other	32.3	20.7	peak 7	1930.938	−94.079	138–152	TSKRPRLVWTPQLHK
					peak 8	1946.958	−93.288	153–169	RFVDVVAHLGIKNAVPK

^a^**C**, Carbamidomethyl modification.

**Table 2.** Peptide identification for haem-binding proteins in *Cy. merolae*.

description	protein accession numbers	closest homologue of *A. thaliana*	putative localization	protein molecular size (kDa)	digest matches score (Mascot score)	peak no.	meas. *m/z*	ppm	positions	peptide sequence^a^
starch-associated protein R1	CMT547C	At1g10760	cytosol	176.9	31.6	peak 2	569.331	−80.15	1475–1479	ALIPR
						peak 3	842.528	−34.41	1532–1538	ILSKIGK
						peak 5	944.552	−5.692	85–91	KARIV**C**R
						peak 7	1808.882	−19.997	1460–1474	STGQVRV**C**NYPSKTK
						peak 9	2251.172	13.135	904–924	AELMASPQGALEFSFLIAEAR
mutS family DNA mismatch repair protein MSH5	CMN192C	At3g20475	nucleus	98.5	31.2	peak 2	569.331	−80.178	238–242	VLPAK
						peak 8	1851.882	−75.502	275–289	KIREILTQPI**C**DPAR
						peak 9	2251.172	7.024	427–446	LALESLDSFLESVAQSEKSR
						peak 10	2254.161	30.957	16–35	QSLIDETDGGEEIFLMTTVR
hypothetical protein	CMS174C	None	extracellular?	35.7	29.8	peak 4	870.56	79.731	83–89	RQNSLPR
						peak 7	1808.882	−3.471	202–218	TTSAVAAQRGYSTPDQR
						peak 8	1851.882	−85.814	186–201	AETVHQRRLPHAPALR
cystathionine beta-synthase	CMS037C	At2g20430	chloroplast	56.4	26.9	peak 4	870.56	21.87	125–132	LLGAEIVR
						peak 7	1808.882	−7.59	191–209	VDVFVAGAGTGGTITG**C**AR
						peak 9	2251.172	6.618	313–331	TDVVVVLILPDS**C**RNYMSK
nuclear receptor co-repressor/HDAC3 complex subunit	CML100C	At5g67320	nucleus	60.7	25.7	peak 4	870.56	66.815	250–256	QRRAPSR
						peak 6	995.648	25.975	115–122	ALVNRPKR
						peak 9	2251.172	65.211	78–98	RSTS**C**EENGALAPETVSSADK
probable leucine aminopeptidase	CMH153C	At3g59760 At4g14880	cytosol	68.9	24.5	peak 3	842.528	22.198	309–316	AIIGEALR
						peak 8	1851.882	−38.367	338–353	LVEMHFPLPEGRSPSR
						peak 10	2254.161	−15.379	317–337	TANFPQIYAVGRAAASRHAPR
Asp–Glu–Ala–Asp (DEAD)-box ATP-dependent RNA helicase	CML137C	At2g24200	nucleus	66.4	24.4	peak 4	870.56	−19.981	165–172	VAVLSLLR
						peak 5	944.552	11.224	391–398	LLAEEISK
						peak 8	1851.882	−53.312	57–73	IQSVPGVPQELADTLER
similar to GTPase-activating protein	CMJ230C	At4g15850	vesicle	55.0	23.3	peak 3	842.528	−34.393	14–20	ALLTRLR
						peak 8	1851.882	−79.014	2–18	ESAQPLPLAESRALLTR
						peak 9	2251.172	2.02	502–520	NVSKRLSSTVTELFEDLDR
hypothetical protein	CMB149C	At4g17890	chloroplast	10.2	22.5	peak 1	525.298	−5.276	49–52	GHRR
						peak 2	569.331	92.113	81–85	HAGDK
similar to nuclear pore complex protein NUP107	CMC129C	At3g14120	nucleus	92.8	22.2	peak 5	944.552	33.606	167–173	VLEWLER
						peak 10	2254.161	−4.815	14–34	SALPSLAAYSDVSEHVEPLIR
						peak 11	2440.286	46.781	203–224	WGLSLTNGTAFDMDAPFRGDLR

^a^**C**, carbamidomethyl modification.

Subcellular localizations of *A. thaliana* candidate proteins (table 1) were predicted based on gene ontology cellular localization in the TAIR database (https://www.arabidopsis.org/). For *Cy. merolae* candidate proteins (table 2), subcellular localizations were predicted by the TargetP program [29], as well as from those of closest homologues of *A. thaliana*. Among candidate proteins, four proteins from *A. thaliana* and three proteins from *Cy. merolae* were predicted to be plastid-localized. In both organisms, half of the candidates were occupied by putative nucleus-localized proteins (five proteins in *A. thaliana* and five proteins in *Cy. merolae*). It is interesting to note that nuclear proteins with similar functions were obtained from either or both organisms (see below)*.* Others were hypothetical, cytosolic, mitochondrial or vesicle proteins.

### (b) Plastid-localized candidate proteins

Among the seven identified plastid-localized proteins, we are interested in the *A. thaliana* ABC transporter (At5g19410), which corresponds to ABCG23, as an energy-dependent transport mechanism is required for moving hydrophilic haem through or out of the lipid bilayer [11]. In *A. thaliana*, there are 129 genes encoding the ABC transporter superfamily and ABCG23 is one of 29 members of the WBC subfamily [30] and is a plastid-envelope localized half-molecule type of ABC transporter [31].

Another interesting protein family are the pentatricopeptide repeat (PPR) proteins. In *A. thaliana*, one plastid-localized PPR protein (At3g09650) is identified. In plants, most PPR proteins are supposed to bind RNA with sequence-specific manner and functions in post-transcriptional processes, including RNA editing, RNA splicing, RNA cleavage and translation [32]. At3g09650, corresponds to HCF152, and is involved in the processing of the chloroplast p*sbB-psbT-psbH-petB-petD* transcript unit [33]. Concerning haem regulation, we recently found that GUN1, which is a PPR protein with a small MutS-related (SMR) domain and is the central integrator of retrograde signalling [34], binds to haem and modulates tetrapyrrole biosynthesis [35].

### (c) Nuclear-localized candidate proteins

Identification of haem-binding nuclear-localized proteins suggests that produced haem is actually transferred and functions in the nucleus in *A. thaliana* and *Cy. merolae*. In this study, we are focused on four functional groups that were identified from either or both organisms.

Haem is known to bind to transcription factors in yeast and mammalian cells [11]. Among identified nuclear proteins, two proteins from *A. thaliana* were transcription factors. At1g27660 belongs to basic/helix–loop–helix (bHLH) superfamily proteins [36]. In the *A. thaliana* genome, 147 bHLH encoding genes have been identified and At1g27660 is assigned as bHLH110, although its function is unknown. Another transcription factor is At5g59570, which corresponds to BROTHER OF LUX ARRHYTHMO (BOA), a component of the circadian clock [37].

The second group is Asp–Glu–Ala–Asp (DEAD)-box ATP-dependent RNA helicases (DBRHs): one protein from *A. thaliana* (At2g35920) and one protein from *Cy. merolae* (CML137C). The DBRH family participates in broad aspects of RNA metabolism, such as transcription, translation, RNA decay and miRNA processing. This is also involved in cell cycle regulation, tumorigenesis, apoptosis, cancer development and viral infection [38], although its physiological function in plants and algae are poorly known.

The third group contains components of nuclear pore proteins: PAUSED (PSD) (At1g72560) from *A. thaliana* and NUP107 homologue (CMC129C) from *Cy. merolae*. In *A. thaliana*, *PSD* encodes an orthologue of exportin-T, which mediates the nuclear transport of tRNA in yeast and mammals [39]. A null *psd* mutant of *A. thaliana* showed defect in various developmental events [39]. NUP107 is localized to the nuclear rim and is an essential component of the nuclear pore complex.

The fourth group contains components of histone deacetylase (HDA): SRT1 (At5g55760) and HDA3 complex subunit (CML100C). HDA removes an acetyl group from Lys residues of histone, resulting in the histone wrapping DNA more tightly that represses the gene expression from the removed chromatin region. SRT1 is involved in the sirtuin family. In humans, SRT1 functions in ageing and metabolism [40]. In *A. thaliana*, it is reported that SRT1 negatively regulates stress tolerance and glycolysis but stimulates mitochondrial respiration through interaction with cMyc-binding protein 1 (AtMBP-1) [41]. The closest homologue of CML100C in *A. thaliana* is At5g67320 corresponding to a WD40 protein HOS15, which interacts with HDA9 to repress transcription of the GIGANTIA-mediated photoperiodic flowering pathway [42].

### (c) Other candidate proteins

Among the remaining proteins, we are interested in a homologue of GTPase-activating protein (CMJ230C). The closest homologue in *A. thaliana* is At4g17890 corresponding to AGD8, a member of the ADP-ribosylation factor 1 (Arf) GTPase-activating proteins (GAP) domain. AGD8 is involved in COP1 vesicle formation for ER to Golgi transport and vice versa. AGD8 is Glo3-type ArfGAP and required for the maintenance of Golgi morphology along with its closest homologue AGD9 [43].

### (d) Characterization of haem-binding proteins

To verify whether candidate proteins actually bind to haem, we produced several recombinant proteins for the haem-binding assay. We chose soluble globular proteins for *in vivo* expression in *E. coli*: HCF152 (At3g09650), SRT1 (At5g55760), GTPase-activating protein (CMJ230C) and HDA3 complex subunit (CML100C).

**Figure 2.**
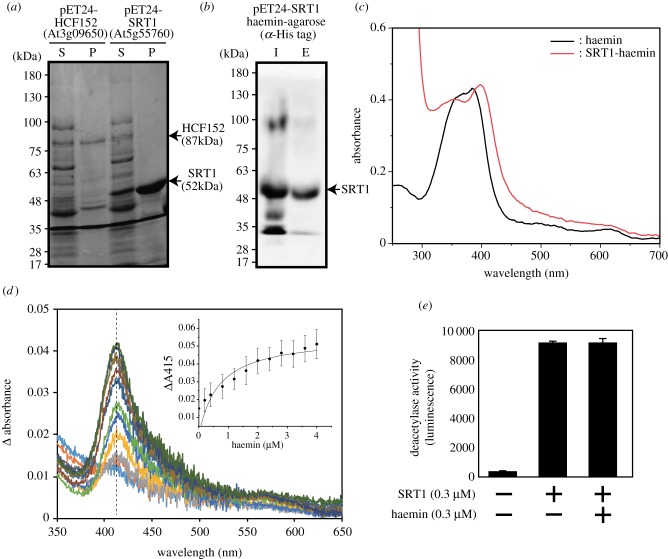
*Arabidopsis thaliana* SRT1 binds to haem. (*a*) Expression of recombinant proteins of *A. thaliana* HCF152 (At3g09650) and SRT1 (At5g55760) in *E. coli*. After induction by IPTG, cell extract was separated into soluble (S) and precipitate (P) fractions by centrifugation. Proteins were separated by SDS–PAGE and visualized by Coomassie Brilliant Blue staining. (*b*) Soluble fraction containing SRT1 was inputted (I) into haemin-agarose beads. After extensive washing, bound proteins were eluted (E) with the SDS sample buffer. The recombinant SRT1 protein was detected by western blotting with polyclonal His-tag antibodies. (*c*) By using N-terminal His-tag, recombinant SRT1 protein was purified in homogeneity. Absorption spectra of haemin solution (black) and haemin-SRT1 complex (red). Equal molar concentration (4 µM) of haemin and purified SRT1 were mixed for measurement. (*d*) Differential UV–visible spectra of haemin (0–4 µM) in the presence of SRT1 (1 µM). (Inset) Plot of the absorbance value at 415 nm (dashed line) of haemin as a function of haemin concentration. Data were fitted with nonlinear regression assuming one-site binding. (*e*) Effects of haemin on the sirtuin activity of SRT1. SRT1 showed the sirtuin activity, but addition of equal molar of haemin had no effect on the activity.

**Figure 3.**
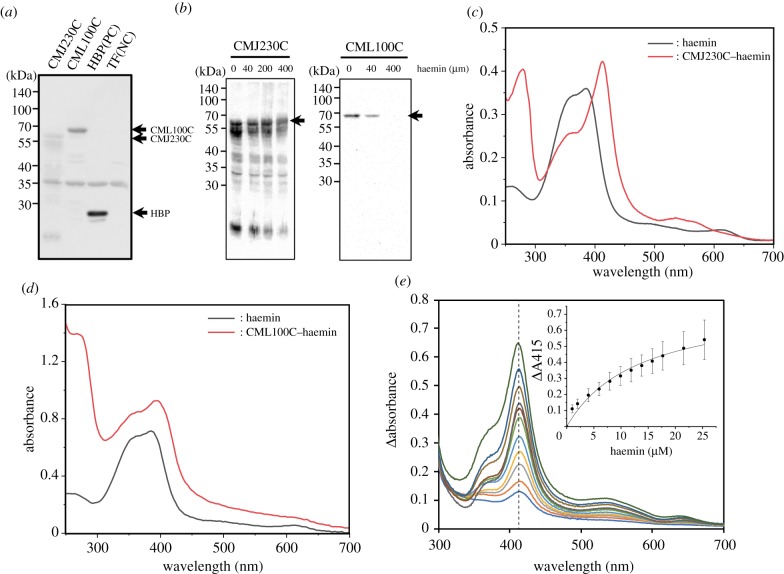
*Cyanidioschyzon merolae* GTPase-activating protein and HDA3 complex subunit bind to haem. (*a*) Soluble fractions of GTPase-activating protein (CMJ230C) and HDA complex subunit (CML100C) were inputted (I) into haemin-agarose beads. After extensive washing, bound proteins were eluted (E) with the SDS sample buffer. The recombinant proteins were detected by western blotting with polyclonal His-tag antibodies. *Arabidopsis thaliana* p22HBP and *E. coli* TF proteins were used as positive and negative controls, respectively. (*b*) Competitive assay of haem binding. Indicated concentration of haemin solution was mixed with CMJ230C (4 mg ml^−1^) and CML100C (1 ml ml^−1^) and subjected to haemin-agarose beads. Eluted proteins were detected by western blotting with polyclonal His-tag antibodies. (*c*) Absorption spectra of haemin solution (black) and haemin–CMJ230C complex (red). Equal molar concentration (6 µM) of haemin and purified CMJ230C were mixed for measurement. (*d*) Absorption spectra of haemin solution (black) and haemin–CML100C–TF complex (red). To 3.5 µM purified CML100C–TF, threefold concentration (10.5 µM) of haemin and purified SRT1 were mixed for measurement. (*e*) Differential UV–visible spectra of haemin (0–25 µM) as a function of haemin concentration in the presence of CML100C (10 µM). (Inset) Plot of the absorbance value at 415 nm (dashed line) of haemin as a function of haemin concentration. Data were fitted with nonlinear regression assuming one-site binding.

For proteins from *A. thaliana*, full-length cDNA fragments of *HCF152* and *SRT1* were cloned into pET24 in BL21(DE3). After induction, cell lysates were separated into soluble and precipitated fractions by centrifugation. HCF152 protein was expressed as an inclusion body (figure 2*a*) and refolding of the recombinant protein was not successful. Meanwhile, a certain portion of recombinant SRT1 was expressed in soluble fraction as a 52 kDa protein (figure 2*a*). We tested the ability of SRT1 to bind haem using haemin-agarose beads. As shown in figure 2*b*, SRT1 demonstrated haemin-binding activity (figure 2*b*). It is noted that when we tested non-haemoprotein (lysozyme) and haemoproteins (catalase, myoglobin and apo-horseradish peroxidase (HRP)), no binding to haemin-agarose was observed (electronic supplementary material, figure S1). To further characterize the haem-binding property, SRT1 was purified to homogeneity by using His-tag for affinity purification (electronic supplementary material, figure S2). Then, we monitored the haemin binding by absorbance, following the evolution of the Soret peak at 415 nm which appears in the presence of SRT1 (figure 3*e*). The interaction led to an increase in absorbance at this wavelength when increasing the haemin concentration. The absorbance values, plotted in the inset of figure 2*c*, gave a saturation curve from which a *K*_d_ of 0.68 ± 0.40 µM was estimated, assuming one haemin bound per domain. To further analyse the effect of haem on SRT1, we measured the sirtuin activity of SRT1. The obtained SRT1 exhibited the SRT1 activity, but exogenous haemin had no effect on the activity (figure 2*e*).

For proteins from *Cy. merolae*, cDNA of CMJ230C and CML100C were cloned into pETNH in Rosseta II. In this experiment, *A. thaliana* p22HBP protein was used as a positive control and *E. coli* TF protein was as a negative control. Although most proteins were detected in precipitated fractions in both cases, certain portions of recombinant proteins of CMJ230C and CML100C were detected in soluble fractions as 56 and 62 kDa bands, respectively (electronic supplementary material, figure 3). When cell lysates were subjected to haemin-agarose beads, both proteins were detected in eluted fractions (figure 3*a*). Binding profiles of positive (p22HBP) and negative (TF) confirmed the specificity of haem binding. A faint band of CMJ230C may be caused by poor expression in the soluble fraction. In addition, the binding of CMJ230C and CML100C to haemin-agarose was competitively decreased by the incubation with soluble haemin, indicating the specific interaction between CMJ230C/CML100C and haemin (figure 3*b*).

To verify spectral effects of proteins upon haem binding, we purified CMJ230C and CML100C by using His-tag for affinity purification (electronic supplementary material, figure S4). In the case of CML100C, required amounts of soluble purified protein for spectral analysis were only obtained when expressed with the pColdTF system. Thus, CML100C was expressed as a fusion protein of *E. coli* TF (CML100C–TF) with this system and purified, while purified TF was used as a negative control. Similar to *Arabidopsis* SRT1, mixing with equal molar concentration of haemin solution with CMJ230C (6 µM) caused the red-shift of the haemin peak to 412 nm (figure 3*c*). In the case of CML100C–TF, a low concentration of purified protein (3.5 µM) was mixed with threefold molar concentration of haemin solution (10.5 µM), which resulted in a slight red-shift of the haemin peak to 415 nm (figure 3*d*). When equal molar of TF and haemin was mixed, no spectral shift was observed (electronic supplementary material, figure S4*e*), confirming specific haem binding of CML100C. We further monitored the haemin binding by absorbance, following the evolution of the Soret peak at 415 nm which appears in the presence of CML100C. The absorbance values, plotted in the inset of figure 3*c*, gave a saturation curve from which a *K*_d_ of 1.33 ± 0.25 µM was estimated, assuming one haemin bound per domain. These results demonstrated that, as well as *Arabidopsis* SRT1, *Cy. merolae* and CML100C bind to haem with high specificity.

## 4. Discussion

In this study, we performed proteomic analysis of haem-binding proteins in *A. thaliana* and *Cy. merolae* by using haemin-immobilized high-performance magnetic FG beads. As designed [26], FG beads showed extremely low non-specific binding of proteins (figure 1) and we could identify several candidates of haem-binding proteins from both organisms. Interestingly, canonical haemoproteins were not involved in haemin-binding proteins, probably covalently or non-covalently attached haem prevented the binding to proteins. In fact, when we analysed haemoproteins (catalase, myoglobin, apo-HRP) for haemin-agarose assay, no binding was observed (electronic supplementary material, figure S1). It is interesting to note that apo-HRP, which spontaneously binds to haemin to form an active holo-enzyme, did not bind to haemin-agarose. Thus, it is likely that only proteins which can bind to haemin at the surface with substantial specificity can bind to haemin-liganded beads. In addition, already reported haem-binding proteins such as Fbx3, TSPO and p22HBP/SOUL were not included in this list, probably protein binding was dependent on their expression, solubility and affinity to the beads. It should be noted that because many identified proteins had not been annotated as haem-binding proteins, we should be careful to check whether each listed protein is actually binding to haem.

Some candidate proteins were possibly involved in haem transfer. Plastid-envelope localized *A. thaliana* ABC transporter ABCG23 (At5g19410) is a potential candidate protein for haem transport. For haem transfer, *Cy. merolae* GTPase-activating protein (CMJ230C) is another interesting candidate. In *A. thaliana* [24] and *Cy. merolae* [20], ABA-inducible TSPO, which is located in the ER-to-Golgi membrane protein, is involved in haem scavenging. In *Cy. merolae*, ABA-inducible TSPO may decrease in the level of unbound haem that inhibits DNA replication [20]. It is therefore important to characterize how haem trafficking in the ER-to-Golgi membrane system, which is mediated by vesicle transport, occurs. In this sense, further analysis of CMJ230C, together with *Cy. merolae* TSPO (CMS231C), is necessary.

Surprisingly, half of the candidate proteins were nucleus proteins in both organisms, supporting the hypothesis that haem is actually transferred to the nucleus for regulatory and/or signalling purposes in these organisms like animal and yeast cells. Furthermore, identification of nuclear haem-binding proteins with similar functional or localization from either or both organisms may indicate the fundamental function of haem in these organisms. Because the detailed function of candidate proteins has not been elucidated in these organisms, further analysis is still needed for understanding. However, considering the general function of candidate proteins, it is possible that haem is involved in transcription through transcription factors, RNA metabolism through RBDHs and nucleoporins, and epigenetic histone modification through HDAs in *A. thaliana* and *Cy. merolae*.

For transcriptional regulation, haem is known to bind the transcription factor HAP1 in yeast to mediate oxidative stress [44]. In mammals, haem also binds to the basic leucine zipper protein Bach1, which represses genes such as haem oxygenase 1 [45]. Haem also coordinates regulation of metabolism with the circadian clock via the Rev-erb haem sensors [46]. For haem binding, a haem-regulatory motif (HRM) is found in bacteria and eukaryotic systems [47]. In fact, HAP1 and Bach1 contain 7 and 6 HRMs, respectively, while Rev-erb binds to haem with non-classical HRM. In bHLH110 and BOA, we could not detect any classic HRM, so haem may bind to distinct domains if they really bind to haem. It is interesting to note that in mammalian cells, haem biosynthesis is circadian-regulated and several components including Rev-erb bind haem [46]. In tobacco, the FC activity is inversely regulated with that of Mg-chelatase during cyclic photoperiods [48], but the involvement of haem in circadian regulation is totally unknown in plants and algae.

For RNA metabolism, haem is known to bind the haem-binding protein DGCR8 (DiGeorge critical region-8), which is a key miRNA processing enzyme in human cells and requires bound haem for its activity [49]. At present, the functions of candidate DBRHs on RNA metabolism are totally unknown. However, considering the effects of DBRHs [38] and miRNA [50] on cell cycle regulation, testing of the involvement of these components on the haem-dependent cell cycle regulation in *Cy. merolae* [13,20,51] is attractive. Currently, there is no report about haem-dependent regulation on nuclear pore transport. Considering haem is imported into the nucleus through the nuclear pore, it is possible that PSD and NUP107 are involved in haem transport into the nucleus in *A. thaliana* and *Cy. merolae,* respectively.

For epigenetic regulation, histone modification-dependent gene repression is suggested in *A. thaliana* retrograde signalling [52]. In this paper, a chloroplast envelope-bound plant homeodomain transcription factor (PTM) is identified and the proteolytic cleavage of PTM occurs in response to retrograde signals and amino-terminal PTM accumulates in the nucleus, where it activates *ABI4* transcription by histone modifications. However, because recent careful analysis showed no significant involvement of PTM [53] and ABI4 [54] in the retrograde signalling, involvement of haem on such epigenetic regulation needs to be elucidated. In this study, we demonstrated that both *A. thaliana* SRT1 (figure 2) and *Cy. merolae* HDA3 complex subunit (figure 3) have haem-binding activity. Because the sirtuin activity of SRT1 was not affected by haemin, it is possible that haem affects the complex formation, stability and/or localization of SRT1 rather than the SRT1 activity. It is noted that the HDA3 complex subunit itself had no significant HDA activity in our assay. Because exogenously treated haem affected cell cycle regulation in *Cy. merolae* [13,20,51] and global gene expression in *Ch. reinhardtii* [18], analysis of haem-dependent histone modification and transcriptome should be investigated in the future.

By using haemin-immobilized high-performance beads, we have succeeded in identification of novel haem-binding candidate proteins from *A. thaliana* and *Cy. merolae*. As half of the candidates were occupied with nucleus proteins, it is likely that haem functions as an actual signal molecule in these organisms. The identification of nucleus proteins with similar function or localization suggests the fundamental but unknown function of haem, which may lead significant studies in retrograde signalling.

In summary, it has been considered that haem acts as a retrograde signalling molecule in *A. thaliana* and *Cy. merolae*. We recently reported that the major retrograde signalling protein GUN1 can bind haem, activate the FC1 activity and regulate the flow through the tetrapyrrole biosynthesis pathway [35], that supports a role for haem in mediating retrograde signalling and opens up the opportunity to develop a unifying hypothesis for this pathway. Therefore, our comprehensive analysis of haem-binding proteins will significantly contribute for the elucidation of this pathway in the future.

## Supplementary Material

Supplementary materials

## Data accessibility

This article has no additional data.
