# Supplementary material for: Proteomic analysis of haem-binding protein from Arabidopsis thaliana and Cyanidioschyzon merolae
Source: Philos Trans R Soc Lond B Biol Sci. 2020 May 4;375(1801):20190488. doi: 10.1098/rstb.2019.0488 (PMC7209954; doi:10.1098/rstb.2019.0488)
Supplement: Supplementary materials [file rstb20190488supp1.docx]

Supplementary material

Supplemental Fig. 1. Hemin-agarose binding assay of hemoprotein.

One-micro gram of lysozyme (non-hemoprotein), catalase, myoglobin (hemoprotein), and apo-protein of horseradish peroxidase (HRP) were subjected to hemin-agarose beads (H) or non-liganded agarose (A). After extensive washing, bound proteins were eluted by SDS-PAGE sample buffer and separated by SDS-PAGE. Each input (I) was loaded at the center. Proteins were visualized by CBB staining.

Supplemental Fig. 2. Purification of recombinant *A. thaliana* SRT1 protein.

**a.** Elution profile of purified protein. Recombinant protein of *A. thaliana* SRT1 (At5g55760) was expressed in *E. coli*. After induction, soluble fraction containing expressed SRT1 protein was subjected to 1 ml of HisTrap column using AKTA Start apparatus. Protein concentration was monitored by A280. After washing, bound proteins were eluted by linear gradient of imidazole (5-500 mM) and eluted fractions were collected. **b.** CBB staining of SDS-PAGE of eluted fractions. Fractions containing pure SRT1 (fractions 8 to 11) were dialyzed for further analysis.

Supplemental Fig. 3. Expression of recombinant proteins of *C. merolae* in *E. coli*.

**a.** Expression of recombinant proteins of *C. merolae* GTPase activating protein (CMJ230C) and HDA complex subunit (CML100C). After induction of recombinant proteins by IPTG, cell extracts were separated into precipitated (P) and soluble (S) fractions, and analyzed by SDS-PAGE. Arrows indicated expressed recombinant proteins. b. Expression of positive (HBP) and negative (TF) controls. Recombinant proteins of *A. thaliana* p22HBP and *E. coli* TF were induced by IPTG. Cell extracts were separated into precipitated (P) and soluble (S) fractions, and analyzed by SDS-PAGE. Arrows indicated expressed recombinant proteins.

Supplemental Fig. 4. Purification of recombinant *C. merolae* CMJ230C and CML100C proteins.

**a.** Elution profile of *C. merolae* CMJ230C protein expressed in *E. coli*. **b.** CBB staining of SDS-PAGE of eluted fractions. Although degradation products were detected, full-length CMJ230C was obtained as the main band in fractions 7 and 8, which were dialyzed for further spectra analysis. **c.** Elution profile of *C. merolae* CML100C-TF protein expressed in *E. coli*. **d.** CBB staining of SDS-PAGE of eluted fractions. By using pColdTF system, CML100C was expressed as *E. coli* TF fusion protein (CML100C-TF). Purified CML100C-TF was obtained in fractions 4 to 7, which were dialyzed for further spectra analysis. **e.** Absorption spectra of hemin solution (black) and hemin-TF mixture (red). Equal molar concentration (6 µM) of hemin and purified TF were mixed for measurement. Note no spectral shift was occurred by mixing *E. coli* TF with hemin solution.

**Supplemental Fig. 1**

**Supplemental Fig. 2**

**Supplemental Fig. 3**

**
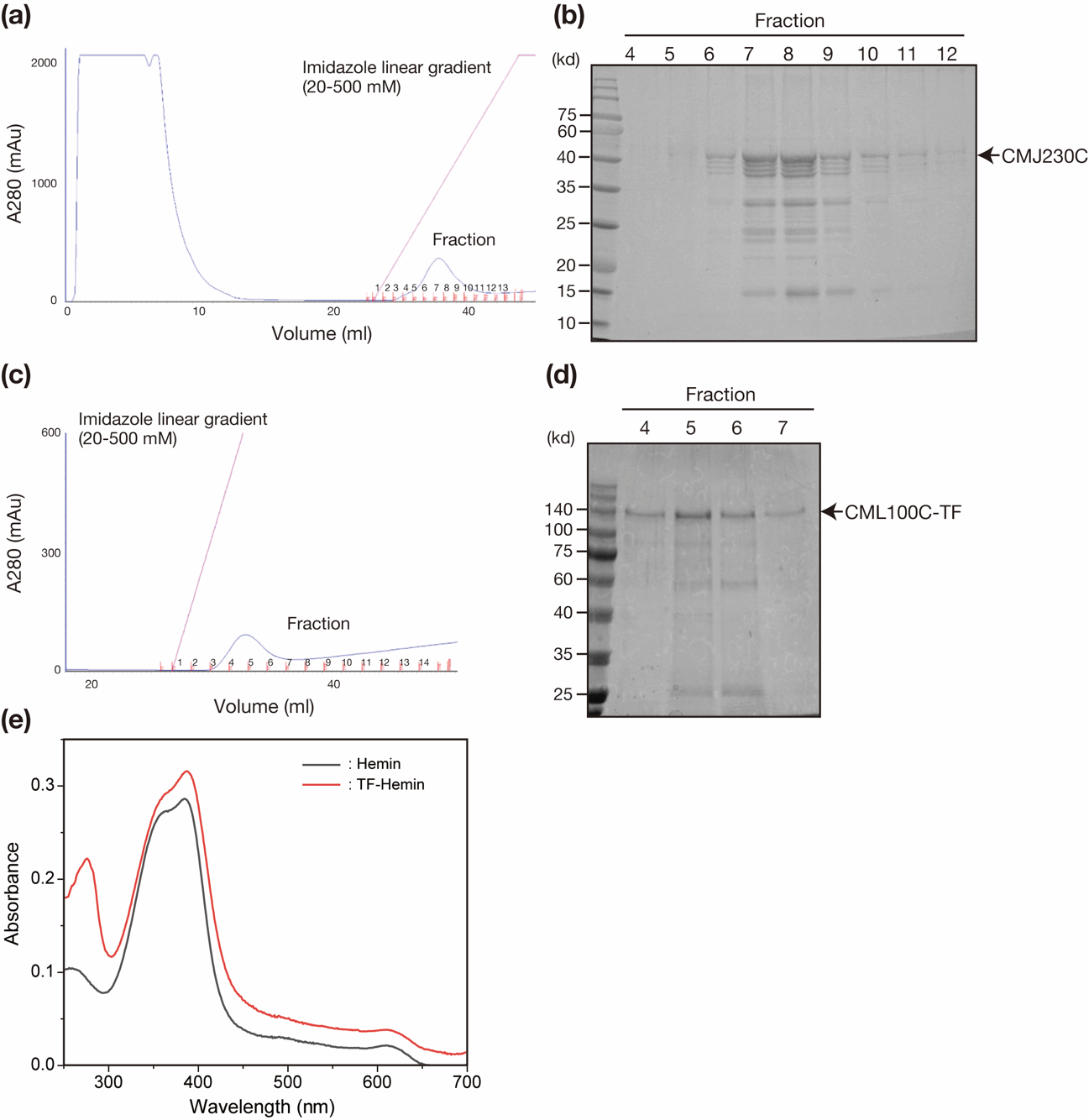
**

**Supplemental Fig. 4**

**Supplemental Table 1. Oligonucleotide DNA primers used in this study.**

| **Primer name** | **Sequence** | **Note** |
| --- | --- | --- |
| pET24f | CTCGAGCACCACCACCACCACCACTGAGATCC | Primer set for amplification of pET24 vector |
| pET24r | CATATGTATATCTCCTTCTTAAAGTTAAAC |  |
| HCF152f | GGAGATATACATATGAACATTCTCCGACCTCCG | Primer set for amplification of *HCF152* |
| HCF152r | GTGGTGGTGCTCGAGGTCTTCTCTTGGACCTAACTTCC |  |
| SRT1f | GGAGATATACATATGTCTTTAGGTTACGCAGAG | Primer set for amplification of *SRT1* |
| SRT1r | GTGGTGGTGCTCGAGTGCCTTGGTTTCTTCTGCC |  |
| pETNHf | CTCGAGCACCACCACCACCACCACTGAGATCCGGCTGC | Primer set for amplification of pETNH vector |
| pETNHr | GAATTCATGATGATGATGATGATGCATATGTATATCTC |  |
| CMJ230Cf | TCATCATCATGAATTCATGGAGTCCGCACAACCT | Primer set for amplification of *CMJ230C* |
| CMJ230Cr | GGTGGTGGTGCTCGAGTTAAGCGCTACGGTCCAG |  |
| CML100Cf | TCATCATCATGAATTCATGTCGAACACTATCACGTC | Primer set for amplification of *CML100C* |
| CML100Cr | GGTGGTGGTGCTCGAGTTACATGCGGAGGTCGGT |  |
